# Supplementary material for: Micellar Hyaluronidase and Spiperone as a Potential Treatment for Pulmonary Fibrosis
Source: Int J Mol Sci. 2021 May 25;22(11):5599. doi: 10.3390/ijms22115599 (PMC8198946; doi:10.3390/ijms22115599)
Supplement: Supplementary file 1 [file ijms-22-05599-s001.zip › ijms-1223786-supplementary.pdf]

Micellar Hyaluronidase and Spiperone as a Potential Treatment for Pulmonary Fibrosis

Evgenii Skurikhin [1,*], Pavel Madonov [2], Olga Pershina [1], Natalia Ermakova [1], Angelina Pakhomova [1], Darius Widera [3], Edgar Pan [1], Mariia Zhukova [1], Lubov Sandrikina [1], Andrey Artamonov [2] and Alexander Dygai [1,4]

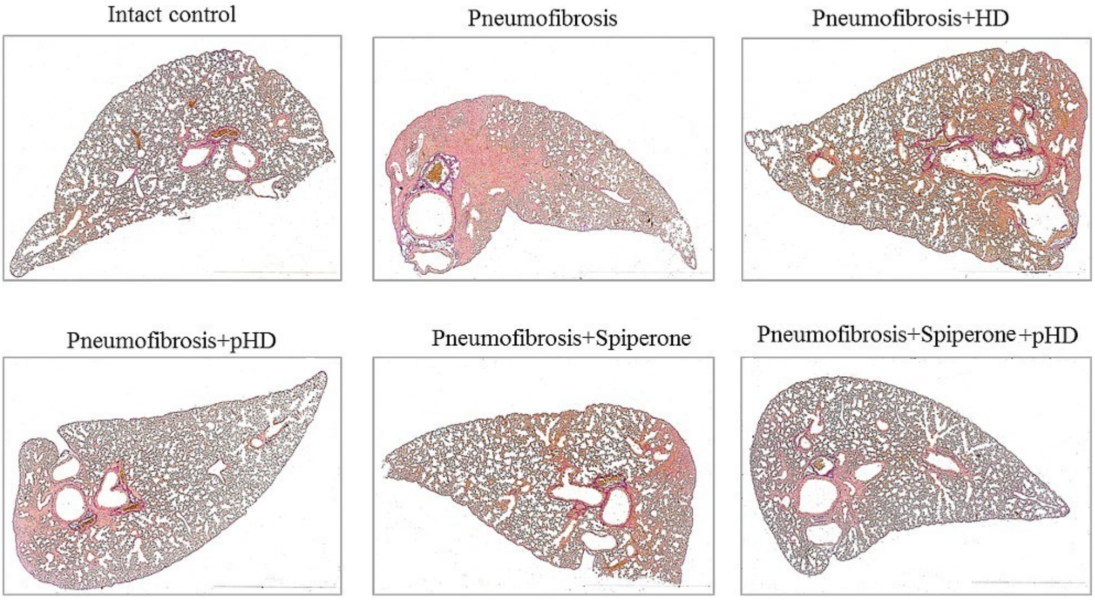

Figure S1. Photomicrographs of left lung sections (cross section of lung) obtained from male C57BL/6 mice on d21. Groups: Intact control; mice with bleomycin-induced pneumofibrosis (Pneumofibrosis); mice with bleomycin-induced pneumofibrosis treated HD (Pneumofibrosis+HD); mice with bleomycin-induced pneumofibrosis treated pHD (Pneumofibrosis+pHD); mice with bleomycin-induced pneumofibrosis treated Spiperone (Pneumofibrosis+Spiperone); mice with bleomycin-induced pneumofibrosis treated Spiperone+pHD (Pneumofibrosis+Spiperone+pHD). Tissues stained by Van Gieson, scale bar 2000 μm.

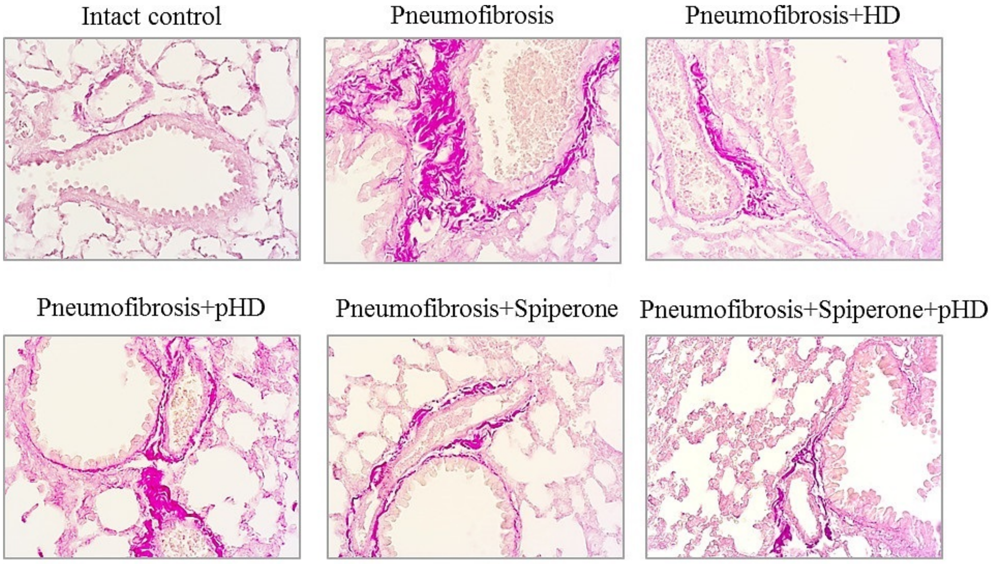

Figure S2. Photomicrographs of left lung sections (middle pulmonary field) obtained from male C57BL/6 mice on d21. Groups: Intact control; mice with bleomycin-induced pneumofibrosis (Pneumofibrosis); mice with bleomycin-induced pneumofibrosis treated HD (Pneumofibrosis+HD); mice with bleomycin-induced pneumofibrosis treated pHD (Pneumofibrosis+pHD); mice with bleomycin-induced pneumofibrosis treated Spiperone (Pneumofibrosis+Spiperone); mice with bleomycin-induced pneumofibrosis treated Spiperone+pHD (Pneumofibrosis+Spiperone+pHD). Tissues stained by Van Gieson, 64X.

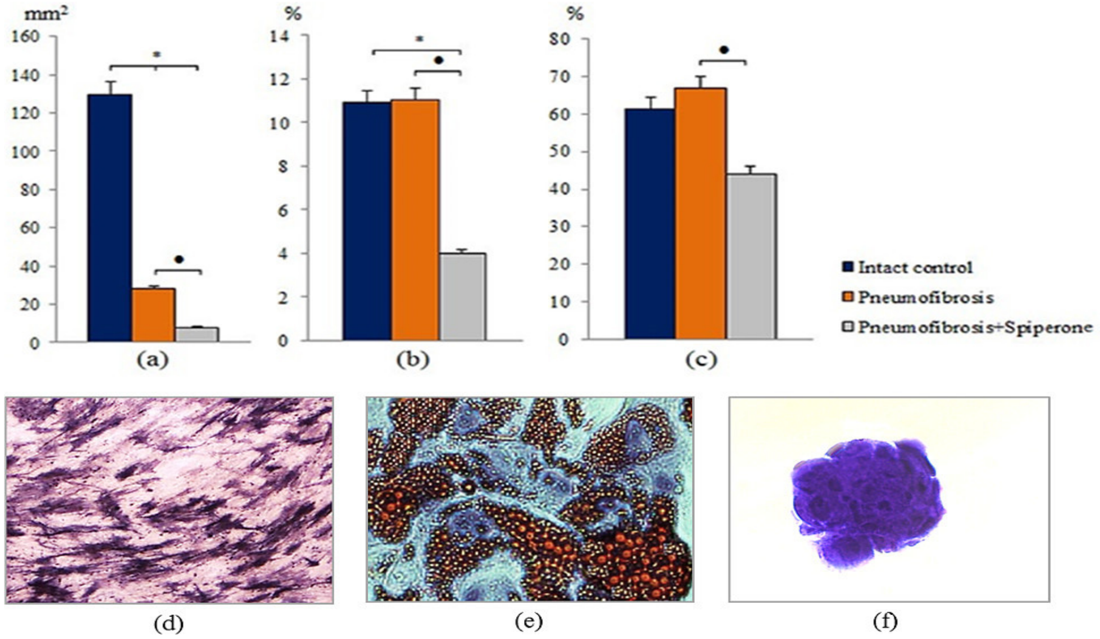

Figure S3. Multilineage differentiation of primary culture adherent cells isolated from the lungs of C57BL/6 mice. Groups: Intact control; mice with bleomycin-induced pneumofibrosis (Pneumofibrosis); mice with bleomycin-induced pneumofibrosis treated Spiperone (Pneumofibrosis+Spiperone). Osteogenic differentiation (**a**, **d**); adipogenic differentiation (**b**, **e**); chondrogenic differentiation (**c**, **f**). The ordinate axis: area of calcium deposits (**a**); cells with lipid inclusions (% of total number of mononuclears) (**b**); cells containing sulfated proteoglycans (% of total number of mononuclears) (**c**). * – $p<0.05$ significance of difference compared with intact control group; ● $p<0.05$ significance of difference compared with the Pneumofibrosis group. Photomicrograph of osteogenic differentiation (**d**). Osteogenic differentiation was demonstrated by the presence of mineralized nodules stained black/purple with von Kossa staining. 400X. Photomicrograph of adipogenic differentiation (**e**). Adipogenic-induced cells contained single lipid droplets, stained orange by oil red O. 400X. Photomicrograph of chondrogenic differentiation (**f**). Chondrogenic differentiation was assessed histologically by demonstrating the presence of cartilage-related matrix components in the specimens. Chondrogenic-induced pellets from all assayed cell sources showed intense purple metachromasia in toluidine blue staining, indicating a high content of sulfated proteoglycans. 100X.

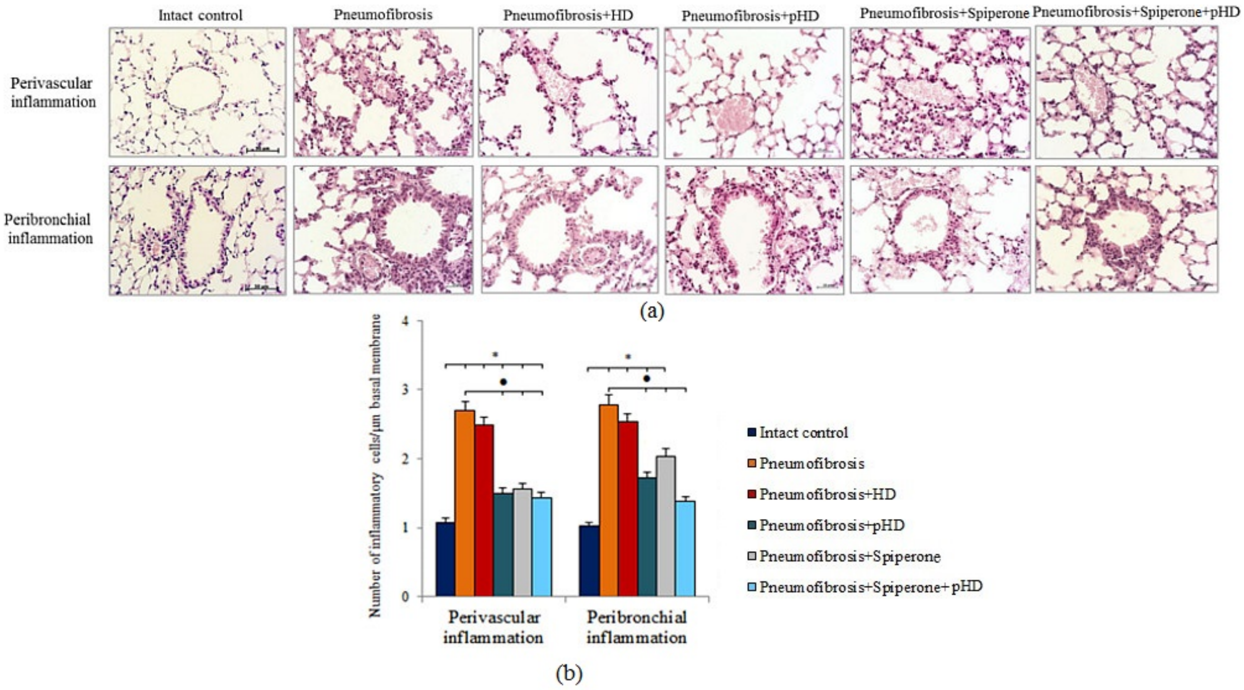

Figure S4. Peribronchial and perivascular inflammation in the lung tissue. (**a**) Photomicrographs of left lung sections (middle pulmonary field) obtained from male C57BL/6 mice on d21. Tissues stained with hematoxylin-eosin, scale bar 10μm. Groups: Intact control; mice with bleomycin-induced pneumofibrosis (Pneumofibrosis); mice with bleomycin-induced pneumofibrosis treated HD (Pneumofibrosis+HD); mice with bleomycin-induced pneumofibrosis treated pHD (Pneumofibrosis+pHD); mice with bleomycin-induced pneumofibrosis treated Spiperone (Pneumofibrosis+Spiperone); mice with bleomycin-induced pneumofibrosis treated Spiperone+pHD (Pneumofibrosis+Spiperone+pHD). (**b**) Quantification of histological inflammation (total inflammation index (number of inflammatory cells/μm of basement membrane). * – $p<0.05$ significance of difference compared with intact control group; ● $p<0.05$ significance of difference compared with the Pneumofibrosis group.
